# Supplementary material for: Unraveling the orientation of phosphors doped in organic semiconducting layers
Source: Nat Commun. 2017 Oct 5;8:791. doi: 10.1038/s41467-017-00804-0 (PMC5629203; doi:10.1038/s41467-017-00804-0)
Supplement: Supplementary file 1 — Supplementary Information [file 41467_2017_804_MOESM1_ESM.pdf]

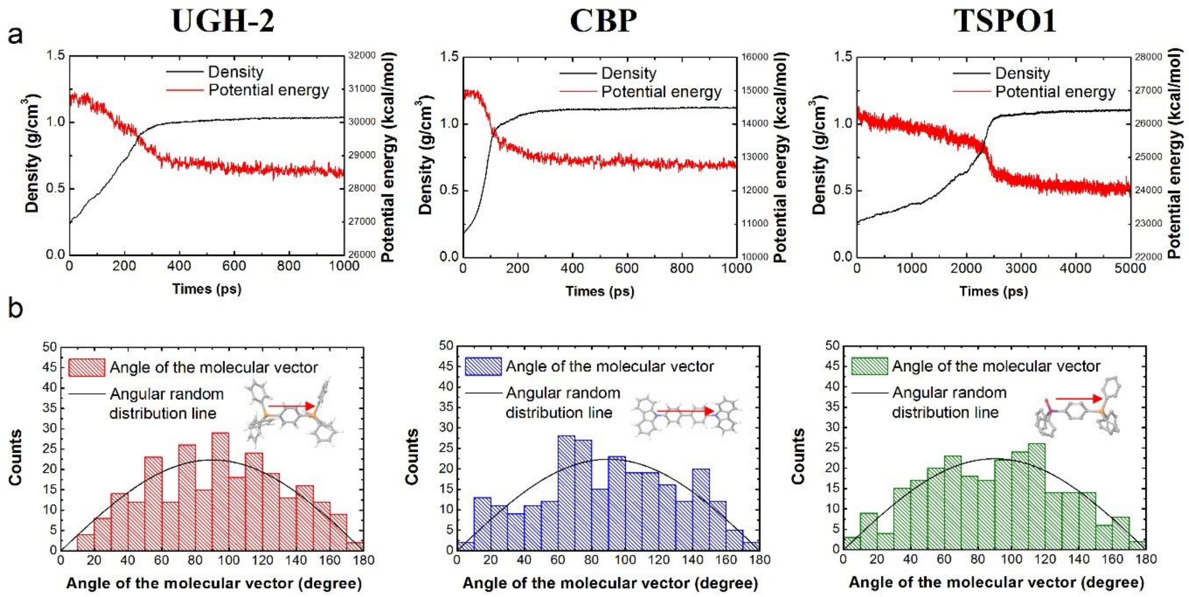

Supplementary Figure 1. Potential energy, volume, and molecular distribution of the organic substrates prepared by MD simulation. (**a**) Change of the density and total potential energy of the substrates consisting of 256-number of UGH-2, CBP, and TSPO1 molecules, respectively, in the simulation of packing the molecules at 300 K and 1 atm. (**b**) Angular distributions of the 256-number of molecular vectors (red arrows) in the substrates. Their distribution followed the random angular distribution line, indicating the amorphous substrates were formed by the MD simulation.

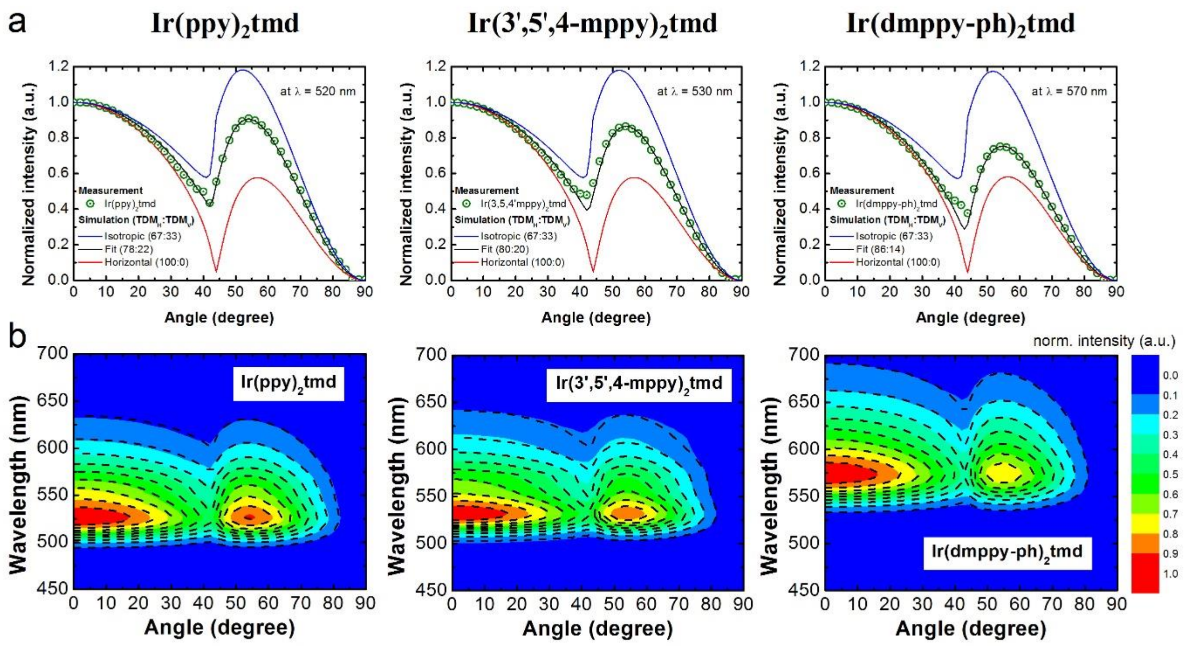

Supplementary Figure 2. Angle-dependent PL analysis of Ir(ppy)2tmd, Ir(3′,5′,4-mppy), and Ir(dmppy-ph)$_2$tmd doped in 30 nm of TSPO1 layers. (**a**) Analysis of the EDO at peak wavelength of each emitter (520 nm, 530 nm, and 570 nm). Measured emission patterns (scatters) are located between the lines with an isotropic orientation (blue) and a fully horizontal orientation (red). The best fitted fractions of the horizontal to vertical emitting dipole moment were 78:22, 79:21, and 85:15, for Ir(ppy)$_2$tmd, Ir(3′,5′,4-mppy), and Ir(dmppy-ph)$_2$tmd, respectively. (**b**) Measured (surfaces) and calculated angular emission spectra (broken lines) having the orientation that have been determined by the analyses at the peak wavelengths.

a

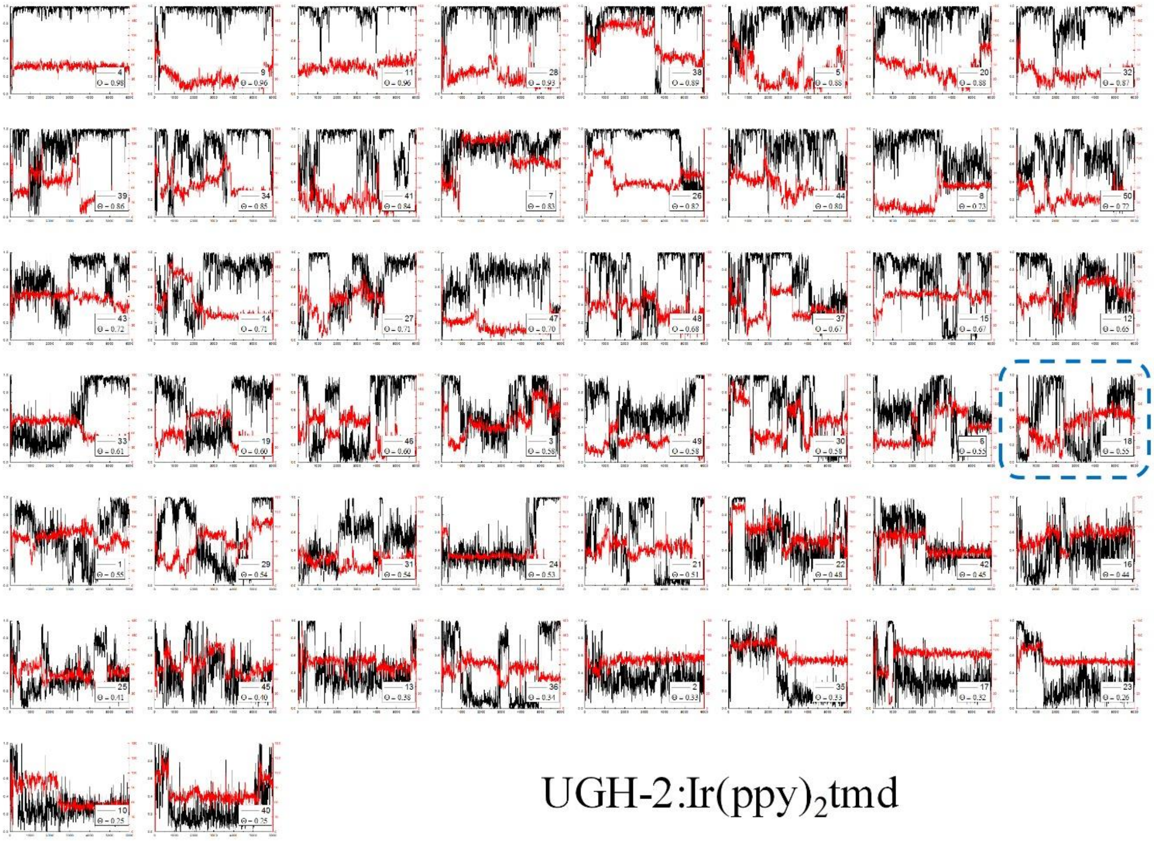

b

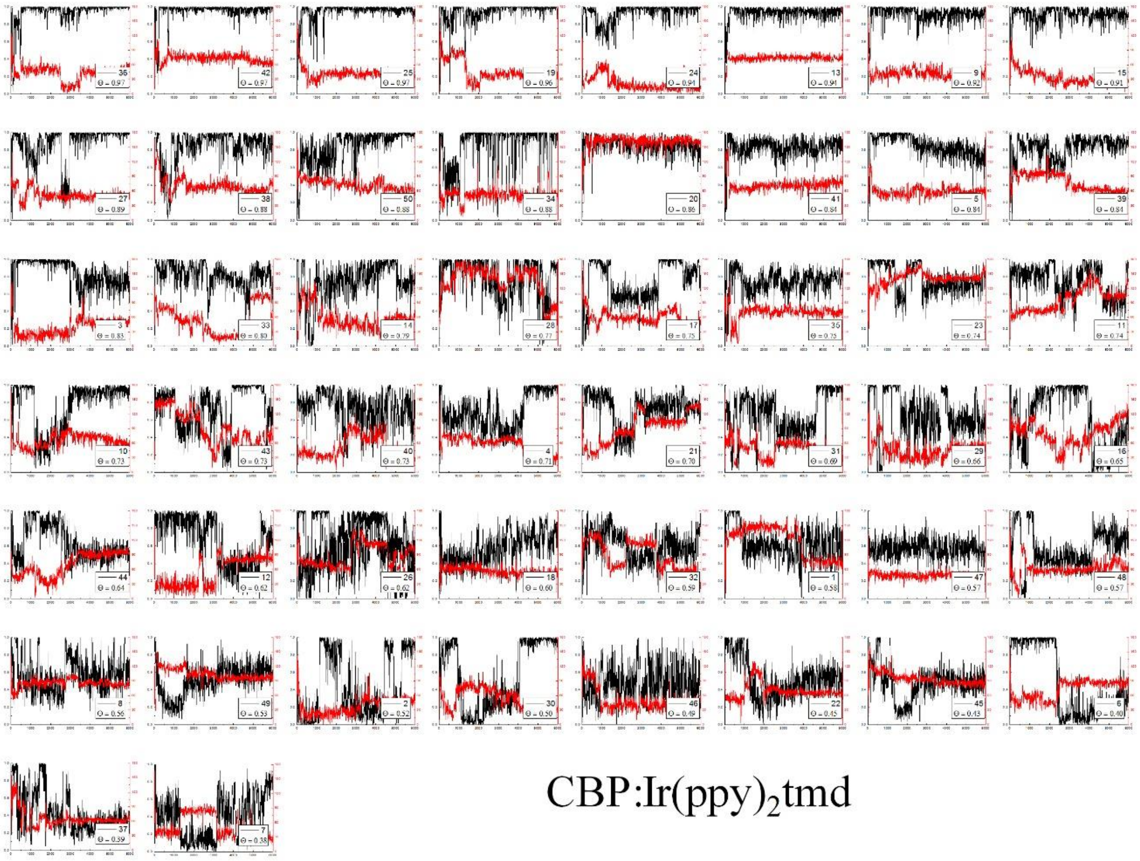

c

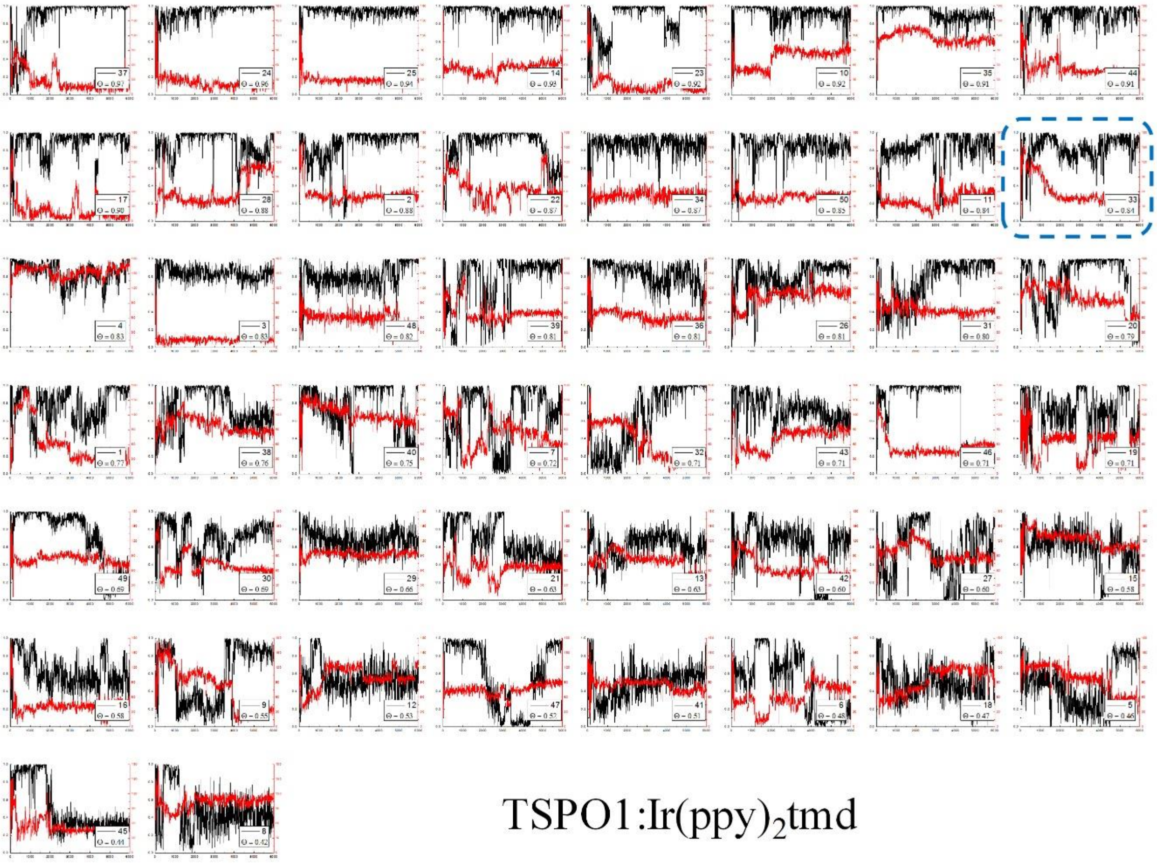

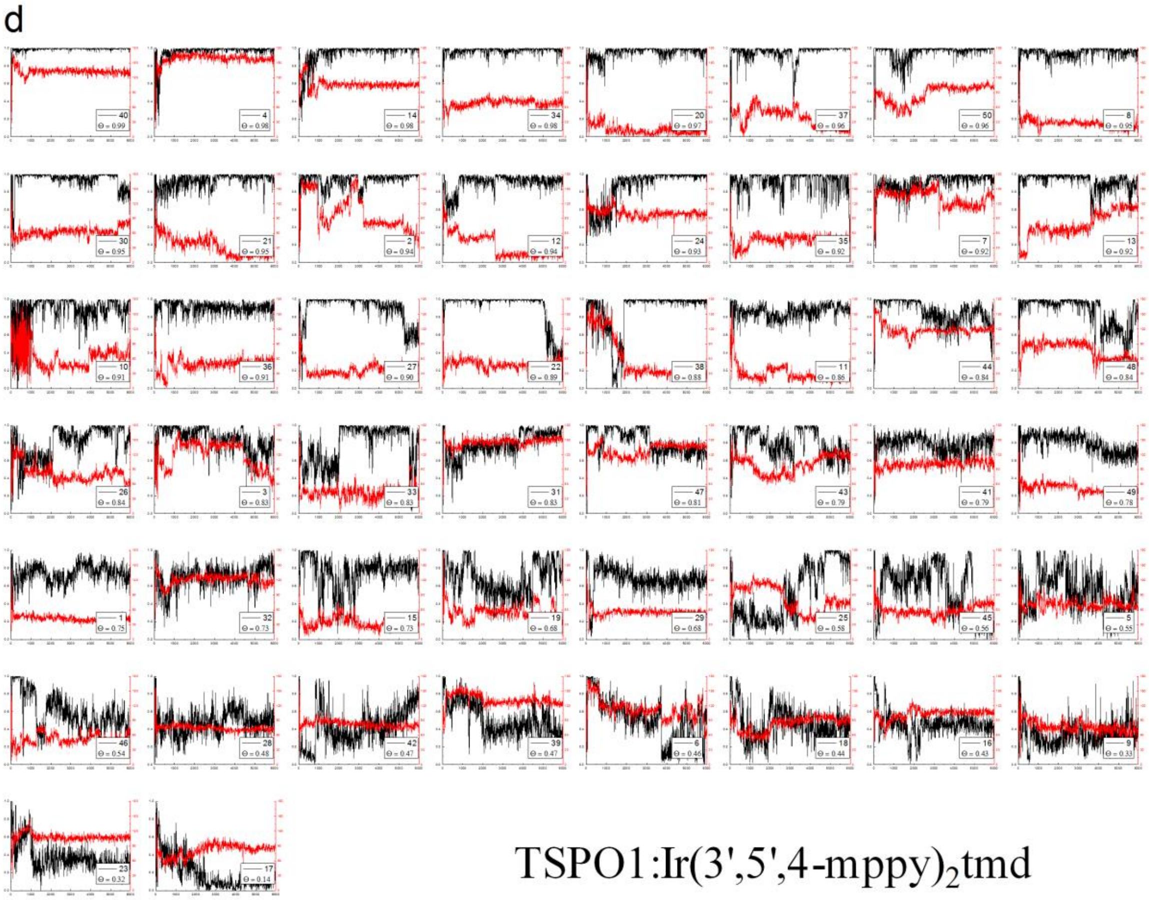
d
TSPO1:Ir(3',5',4-mppy)$_2$tmd

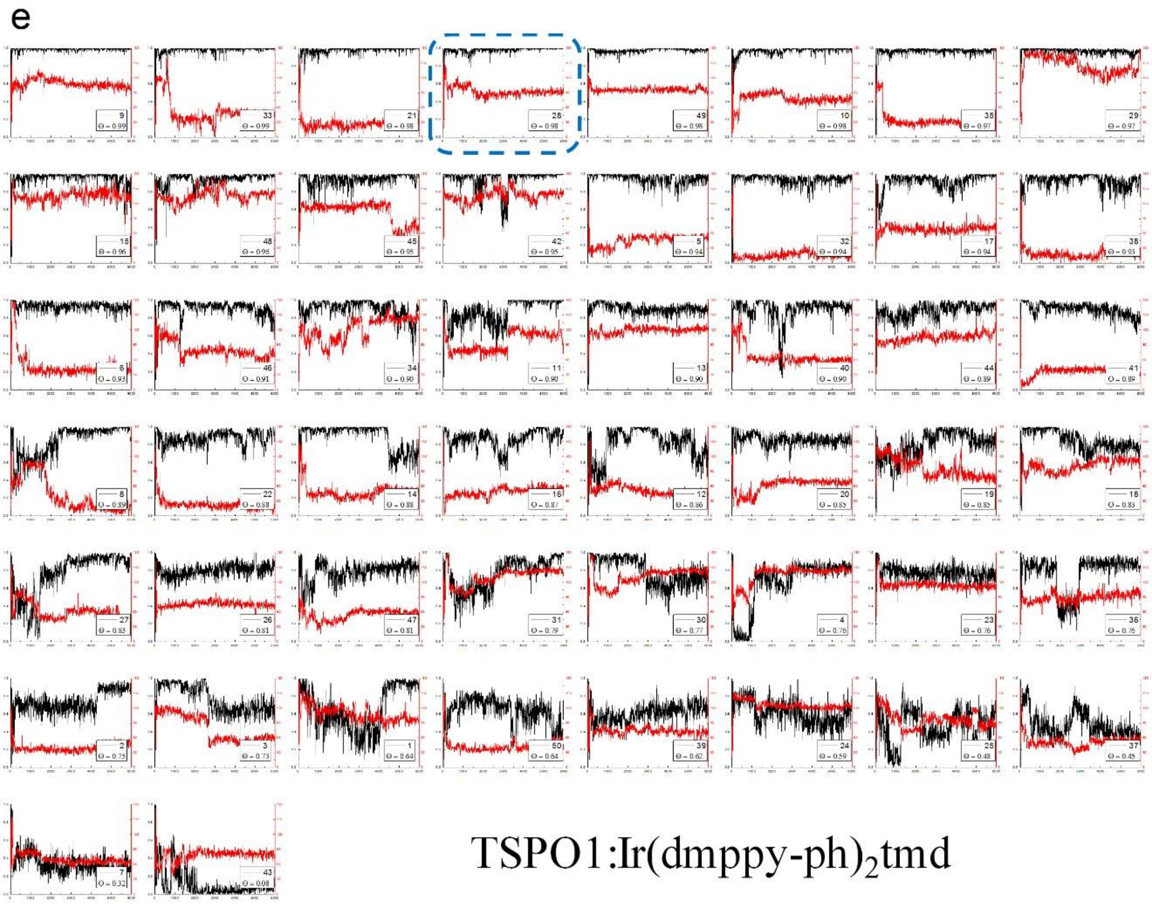

Supplementary Figure 3. 50 trajectories of TDM$_H$ up to 6000 ps in 5 combinations of the host and the dopant. Black and red lines represent the ratio of TDM$_H$ in the range of 0 to 1 and the angle of the C_2 axis of phosphors in the range of 0° to 180°, respectively. Data were arranged in the order of standard deviation between 1000 ns and 6000 ns and the numbers in the figure is the simulation numbers.

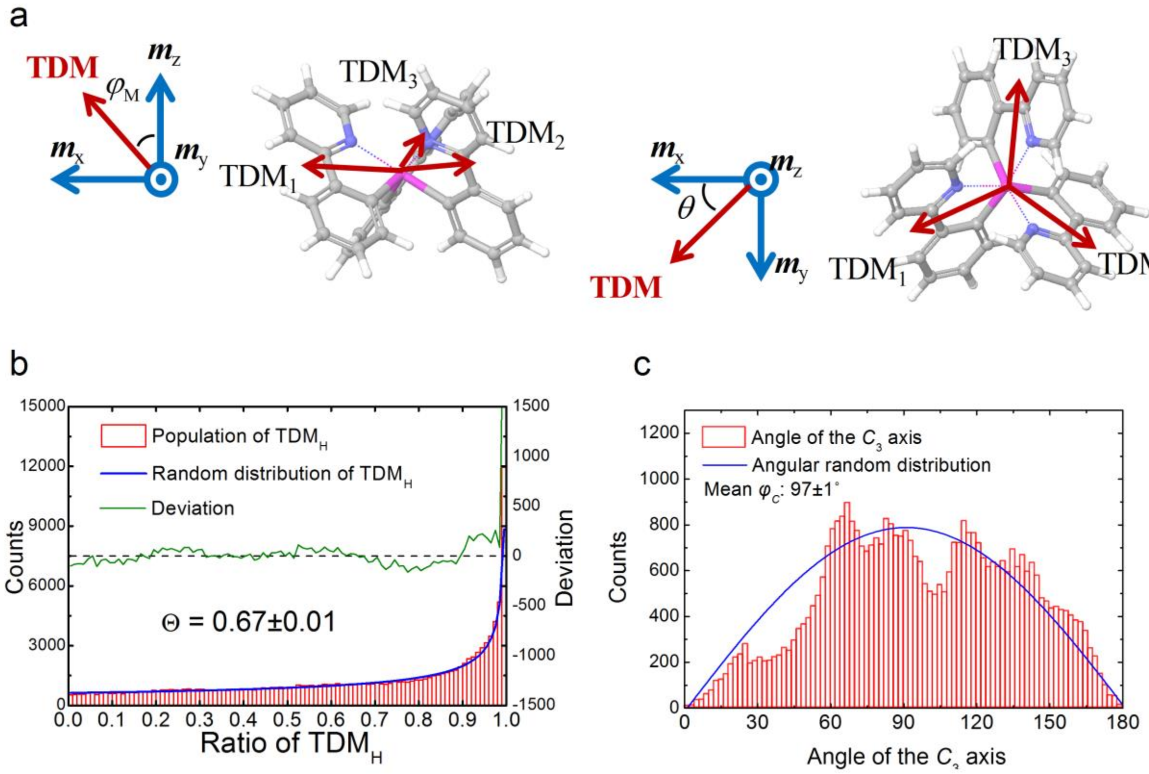

Supplementary Figure 4. Quantum chemical simulation of the TDMs and molecular dynamic simulation of molecular and emitting dipole orientations of Ir(ppy)$_3$. (a) Three triplet TDM vectors of Ir(ppy)$_3$ with a 3-fold rotation symmetry from iridium to three equivalent ppy ligands by ^{3}MLCT. The C_3 symmetry axis toward pyridines from the origin located at the Ir atom was set as $\boldsymbol{m}_z$, the vector normal to the plane including $\boldsymbol{m}_z$ and one of Ir-N vector was set as $\boldsymbol{m}_y$, and $\boldsymbol{m}_x$ was determined by a cross product of $\boldsymbol{m}_y$ and $\boldsymbol{m}_z$ in the dopants. Optimization of the molecular structures were demonstrated using B3LYP method and LACVP** basis set. Spin-orbit coupled time-dependent density functional theory (SOC-TDDFT) calculations were carried out using B3LYP method and DYALL-2ZCVP_ZORA-J-PT-GEN basis set. (b) A histogram of the TDM$_H$ of Ir(ppy)$_3$ with a simulated Θ value. Red bars indicate the population of the phosphor configurations having TDM$_H$ values in steps of 0.01. The blue line is the theoretical line of TDM$_H$ from an arbitrary vector and the green line

represents the deviations between red bars and blue lines. Note that 125100 data were included in the histogram by a product of 41,700 frames and three TDMs. (c) A histogram of the angle of the C_3 axis of Ir(ppy)$_3$ in steps of 2°. The blue line represents an angular random distribution of an arbitrary vector. This histogram includes 41,700 data in total.

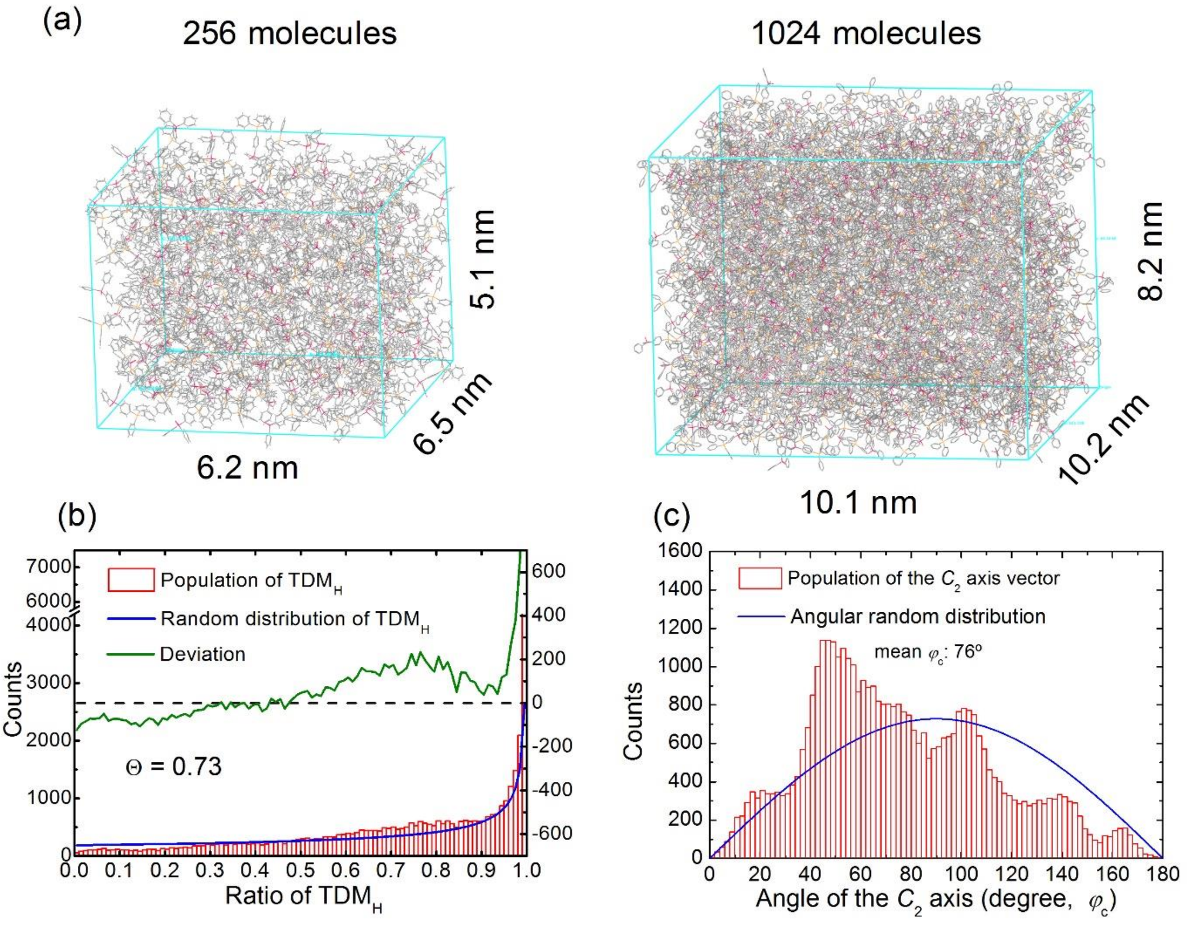

Supplementary Figure 5. Vacuum deposition simulation with a 1024-molecule substrate

(a) Comparison of TSPO1 substrates consisting of 256 and 1024 molecules. (b) Histograms of (a) TDM_H and (b) φ_C demonstrated by the deposition simulation of Ir(ppy)2tmd on the 1024-molecule TSPO1 substrate.

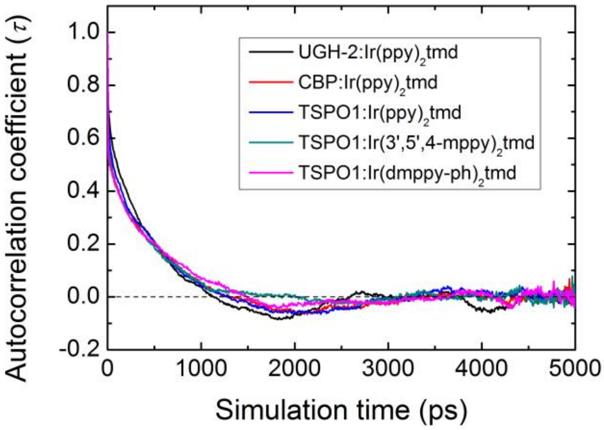

Supplementary Figure 6. Autocorrelation coefficients in the 5 different deposition systems.

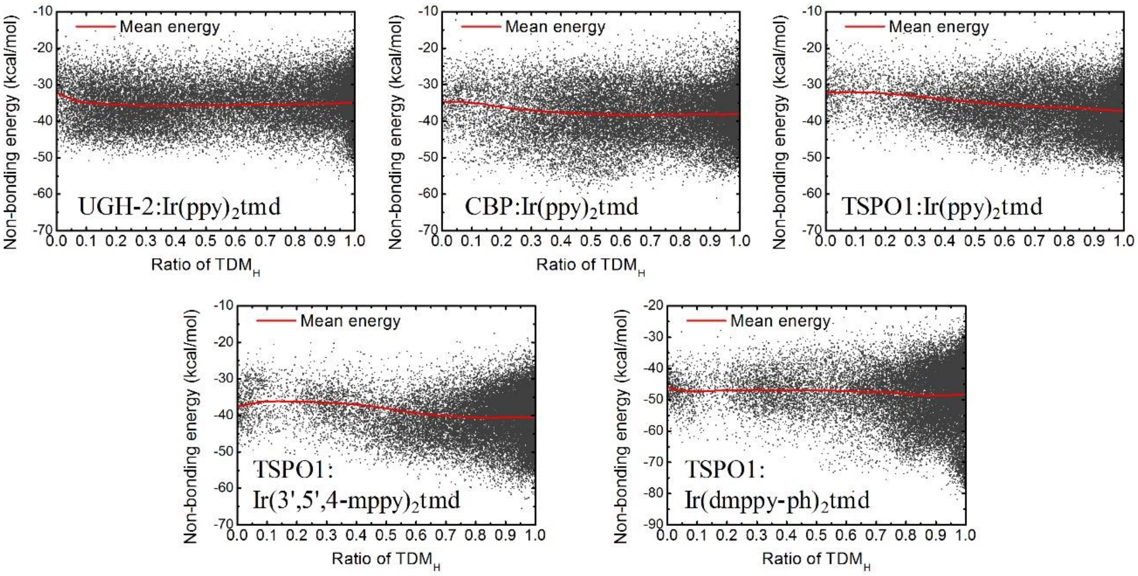

Supplementary Figure 7. Relationship of the emitting dipole orientation in five host-dopant systems. Non-bonded energies of the phosphors located on the surface were calculated by the summation of van der Waals and Coulomb interaction energies with cut-off radius of 0.9 nm of each atom of the phosphors. Red lines represent the mean non-bonded energies as a function of the ratio of TDM_H.

Supplementary Note 1: Preparation of organic substrates by MD

Preparation of the substrate had three steps of the MD simulation after locating 256 molecules in a grid: annealing at 500K at first (NVT, 500 ps) followed by annealing at 300 K (NVT, 200 ps), and finally packing of the molecules at 300K and 1 atm (NPT, 1,000 ps for UGH-2 and CBP, and 5,000 ps for TSPO1). Supplementary Figure 1a shows the trajectories of the density and total potential energy of UGH-2, CBP, and TSPO1 substrates, respectively, at the step of packing. Density and potential energies were converged during the 1,000 ps of simulation for the UGH-2 and CBP substrates and 5,000 ps of simulation for the TSPO1 substrate. The NPT MD simulation produced amorphous solid densities of 1.04, 1.13, and 1.10 g/cm^3 for UGH-2, CBP, and TSPO1, respectively. Consider for example CBP whose simulated density of 1.13 g/cm^3 is in good agreement with the experimental value of 1.18 g/cm^3.[1] Supplementary Figure 1b exhibits the orientation of 256 molecular vectors of UGH-2, CBP, and TSPO1 indicated as red arrows, respectively, consisting the substrates. Their angular distributions were closed to the random distribution line so we concluded that the amorphous organic substrates were successfully prepared.

Supplementary Note 2: Film fabrication and angle-dependent PL measurement

Organic films were fabricated on fused silica substrates by thermal evaporation under a pressure of 5×10^{-7} torr without breaking the vacuum. Rates of the co-deposition were 1 Å/s and thickness of the films were 30 nm. Films were encapsulated using glasses and UV resin after the deposition. EDOs of the organic films were measured by angle-dependent PL analysis. The fused silica substrates were attached to a half-cylinder lens made of fused silica with an index similar densities and matching oil. The attached films were excited by a He-Cd laser (325 nm, CW) and the angular PL spectra escaping out through the lens were measured

by Maya2000 spectrometer (Ocean Optics Inc.). A linear polarizer was used to obtain the *p*-polarized PL patterns. Optical simulation of the luminescence from a thin film was employed to fit the dipole orientation from the PL patterns.[2]

Supplementary Figure 2a shows the angular *p*-polarized emission patterns at peak wavelengths of Ir(ppy)$_2$tmd, Ir(3′,5′,4-mppy), and Ir(dmppy-ph)$_2$tmd doped in 30-nm-thick TSPO1 layers, respectively. Lines are the calculated emission patterns of the *p*-polarized light with an isotropic (blue), fully horizontal (red), and the best-fitted EDO (black). The lower emission intensities of the dyes above 50° represent that the emitter has the larger portion of the horizontal emitting dipole moment. As a result, EDO of Ir(ppy)$_2$tmd, Ir(3′,5′,4-mppy), and Ir(dmppy-ph)$_2$tmd were determined as Θ = 0.78, 0.80, and 0.86, respectively. The orientation values fitted in all the spectral range of the phosphors as shown in Supplementary Figure 2b.

Supplementary Note 3: Deposition simulation of Ir(ppy)$_3$ on CBP substrate

Deposition simulation of Ir(ppy)$_3$ was demonstrated on the pre-organized CBP substrate layer. Ir(ppy)$_3$ is a well-known homoleptic complex exhibiting isotropic emitting dipole orientation (EDO) as doped in organic host layers.[3-4] The identical QM and MD methods described in the main text were employed to analyze the orientation of Ir(ppy)$_3$ deposited on CBP layer using Jaguar[5] and Desmond.[6] The symmetric structure of Ir(ppy)$_3$ results in three equivalent triplet transition dipole moments (TDMs) by ^{3}MLCT with polar coordinates of [φ_M = 87°, θ = 25°], [φ_M = 87°, θ = 145°], and [φ_M = 87°, θ = -95°], with respect to the molecular C_3 axis as shown in Supplementary Figure 4a. Molecular configurations and three TDM vectors of Ir(ppy)$_3$ in the deposition simulation were recorded every 6 ps until reaching 6,000 ps. Supplementary Figure 4b and 4c show the statistical results of the ratio of horizontal TMD (TDM$_H$) and the angle of the C_3 axis of Ir(ppy)$_3$. The distribution of TDM$_H$

follows the random distribution line (blue line in Supplementary Figure 4b) and the ensemble average of Θ=0.67 is in good agreement with the EDO observed by experiments. The angular distribution of the C_3 axis is close to the random distribution line (blue line in Supplementary Figure 4c) as well.

Supplementary Note 4: Deposition simulation of Ir(ppy)$_2$tmd on a 1024-molecule-TSPO1 substrate

To validate if the substrates consisting of 256 molecules appropriately consider intermolecular interactions, we demonstrated the deposition simulation with the larger number of host molecules. Supplementary Figure 5a exhibits the equilibrated 1024-molecule-TSPO1 substrate. Supplementary Figure 5b and 5c show histograms of TDM$_H$ and angle of the C_2 axis of Ir(ppy)$_2$tmd, respectively, deposited on the 1024-molecule substrate. The simulated EDO value (Θ, the ratio of horizontal transition dipole moment) was 0.74 which corresponds to the value of the deposition using the substrate consisting of 256 TSPO1 molecules. The distribution of the C_2 axis vector was broad with the mean angle of 74°, exhibiting no large difference of the molecular distribution with the simulation results using the 256-molecule substrate.

Supplementary Note 5: Autocorrelation times of the deposition simulation

We estimated the integrated autocorrelation times and the effective number of independent samples to validate if the 50-independent-deposition events are sufficient to represent configurations of Ir compounds doped in organic semiconducting layers. The autocorrelation coefficient (τ) as a function of the time lag (t) was calculated by

$$\tau = \frac{1}{50}\sum_{k=1}^{50}\frac{C_A(t,k)}{C_A(0,k)}, \tag{1}$$

where k is the simulation number among the 50 deposition events and C_A is the covariance following $C_A(t) = \langle (A(s+t)-\bar{A})(A(s)-\bar{A}) \rangle$. Supplementary Figure 6 exhibits τ's of the 5 different deposition systems with the x-axis as a product of the lag and the time step of 6 ps. The integrated autocorrelation times (τ_{int}) and the effective number of independent samples (n_{eff}) were calculated using the following formulas:

$$\tau_{\text{int}} = 6\ \text{ps} \times \sum_{t=0}^{t_{\text{final}}} \tau(t), \tag{2}$$

$$n_{\text{eff}} = 50 \times \frac{5000}{2\tau_{\text{int}}}. \tag{3}$$

τ_{int} values were 157, 168, 189, 218, and 208 ps, and n_{eff} values were 765, 715, 638, 557, and 583 for UGH-2:Ir(ppy)$_2$tmd, CBP:Ir(ppy)$_2$tmd, TSPO1:Ir(ppy)$_2$tmd, TSPO1:Ir(3',5',4-mppy)$_2$tmd, and TSPO1:Ir(dmppy-ph)$_2$tmd systems, respectively. The small τ_{int} values compared to the total simulation time of 5000 ps (1000 ps to 6000 ps) indicate no large autocorrelation in the time series data. We concluded that the number of samples is sufficient for the analysis of molecular configuration in the trajectories.

Supplementary References

1. Xiang, H.-F. *et al*. Method for measurement of the density of thin films of small organic molecules. *Rev. Sci. Instrumen*. **78**, 034104 (2007).

2. Moon, C.-K., Kim, S.-Y., Lee, J.-H. & Kim, J.-J. Luminescence from oriented emitting dipoles in a birefringent medium. *Opt. Exp*. **23**, A279–A291 (2015).

3. Liehm, P. *et al.* Comparing the emissive dipole orientation of two similar phosphorescent green emitter molecules in highly efficient organic light-emitting diodes. Appl. Phys. Lett. 101, 253304 (2012).

4. Moon, C.-K., Kim, K.-H., Lee, J. W. & Kim, J.-J. Influence of host molecules on emitting dipole orientation of phosphorescent iridium complexes. *Chem. Mater*. **27**, 2767–2769 (2015).

5. Jaguar 9.2, Schrödinger, LLC, New York, NY, (2016).

6. Desmond Molecular Dynamics System 4.6, D. E. Shaw Research, New York, NY (2016); Maestro-Desmond Interoperability Tools, Schrödinger, New York, NY (2016).
