# Supplementary material for: Unraveling the orientation of phosphors doped in organic semiconducting layers
Source: Nat Commun. 2017 Oct 5;8:791. doi: 10.1038/s41467-017-00804-0 (PMC5629203; doi:10.1038/s41467-017-00804-0)
Supplement: Supplementary file 3 — Description of Additional Supplementary Files [file 41467_2017_804_MOESM3_ESM.pdf]

Description of Additional Supplementary Files

File Name: Supplementary Movie 1
Description: An example of the vacuum deposition simulation of a Ir(ppy)2tmd molecule on a TSPO1 substrate (side view)

File Name: Supplementary Movie 2
Description: An example of the vacuum deposition simulation of a Ir(ppy)2tmd molecule on a TSPO1 substrate (top view)
